# The Aromatic Plant Clary Sage Shaped Bacterial Communities in the Roots and in the Trace Element-Contaminated Soil More Than Mycorrhizal Inoculation – A Two-Year Monitoring Field Trial

**DOI:** 10.3389/fmicb.2020.586050

**Published:** 2020-12-02

**Authors:** Robin Raveau, Joël Fontaine, Mohamed Hijri, Anissa Lounès-Hadj Sahraoui

**Affiliations:** ^1^Université du Littoral Côte d’Opale, Unité de Chimie Environnementale et Interactions sur le Vivant (UCEIV), Calais, France; ^2^Institut de Recherche en Biologie Végétale (IRBV) de l’Université de Montréal, Montreal, QC, Canada; ^3^AgroBioSciences, Mohammed VI Polytechnic University (UM6P), Ben Guerir, Morocco

**Keywords:** phytomanagement, trace elements-contaminated soils, microbiota, *Salvia sclarea*, aromatic plant, essential oils

## Abstract

To cope with soil contamination by trace elements (TE), phytomanagement has attracted much attention as being an eco-friendly and cost-effective green approach. In this context, aromatic plants could represent a good option not only to immobilize TE, but also to use their biomass to extract essential oils, resulting in high added-value products suitable for non-food valorization. However, the influence of aromatic plants cultivation on the bacterial community structure and functioning in the rhizosphere microbiota remains unknown. Thus, the present study aims at determining in TE-aged contaminated soil (Pb – 394 ppm, Zn – 443 ppm, and Cd – 7ppm, respectively, 11, 6, and 17 times higher than the ordinary amounts in regional agricultural soils) the effects of perennial clary sage (*Salvia sclarea* L.) cultivation, during two successive years of growth and inoculated with arbuscular mycorrhizal fungi, on rhizosphere bacterial diversity and community structure. Illumina MiSeq amplicon sequencing targeting bacterial 16S rRNA gene was used to assess bacterial diversity and community structure changes. Bioinformatic analysis of sequencing datasets resulted in 4691 and 2728 bacterial Amplicon Sequence Variants (ASVs) in soil and root biotopes, respectively. Our findings have shown that the cultivation of clary sage displayed a significant year-to-year effect, on both bacterial richness and community structures. We found that the abundance of plant-growth promoting rhizobacteria significantly increased in roots during the second growing season. However, we didn’t observe any significant effect of mycorrhizal inoculation neither on bacterial diversity nor on community structure. Our study brings new evidence in TE-contaminated areas of the effect of a vegetation cover with clary sage cultivation on the microbial soil functioning.

## Introduction

Inorganic and organic soil contamination resulting mainly from anthropogenic practices, is a worldwide concern particularly in urban areas. Contamination with trace elements (TE), especially originating from industrial activities such as smelting and fossil fuel combustion, agriculture and mining activities are among the most frequent inorganic soil contaminants in Europe. They represent more than 30% of the total soil contamination and are found in nearly 2 million hectares of contaminated land (Panagos et al., 2013; Mench et al., 2018). A large number of the contaminated sites remains marginal, due to very-costly remediation methods, large areas to remediate or unprofitable long-term use of the site (Van Liedekerke et al., 2014; Mench et al., 2018). Beside their noxious effects on human health, TE are also likely to affect soil biology and fertility as well as plant development and productivity, representing a major constraint for contaminated agricultural lands (Antoniadis et al., 2017; Gong et al., 2018). It has previously been demonstrated that TE reduce microbial biomass, hence affecting functional diversity and microbial community structure resulting in an impairment of ecosystem services (Ding et al., 2017). Indeed, soil organisms contribute to a wide range of ecosystem services that are essential to the sustainable functioning of natural and managed ecosystems (Delgado-Baquerizo et al., 2016; Saccá et al., 2017). In particular, the soil microbiota of terrestrial plants is of crucial importance, regarding soil fertility, as many microorganisms play a role in biogeochemical cycles, and regarding their contribution to plant nutrition and health (Ding et al., 2017; Misra et al., 2019; Chen et al., 2020). Among them, soil Bacteria are well known for regulating pests and diseases, sequestrating or degrading contaminants, cycling nutrients and mineralizing organic matter as well as being potential biological indicators for soil quality and fertility (Saccá et al., 2017).

Among the potential remediation options, the phytomanagement of contaminated soils, relying on the use of plant species to reduce the risk due to the presence of the contaminant combined with a sustainable and profitable land and biomass use, appears as a promising method to rehabilitate and increase the market value of contaminated sites (Cundy et al., 2016; Mench et al., 2018). It encourages the use of remediation phytotechnologies as part of an integrated site management, providing economic, aesthetic, environmental and social benefits (Burges et al., 2018). Due to the non-biodegradability of TE, the use of phytostabilization, involving the natural ability of plants and their associated microorganisms to immobilize inorganic contaminants such as TE in their rhizosphere, could be particularly relevant. The success of phytomanagement in agricultural contaminated soils will hence depend on the capacity of cultivated plants to grow in contaminated conditions and to develop large amounts of biomass for valorization perspectives (Evangelou et al., 2015). Currently, the research on the valorization of biomass produced on TE-contaminated soil mainly focuses on high biomass plants intended for bioenergy production, such as Miscanthus, maize (*Zea mays* L.), poplar (*Populus* spp.) or willow (*Salix* spp.) (Evangelou et al., 2015; Chalot et al., 2020; Zgorelec et al., 2020). The cultivation of aromatic plants, such as clary sage (*Salvia sclarea* L.), belonging to family Lamiaceae, could nonetheless represent a profitable and innovative alternative for the reconversion of agricultural contaminated soils. In particular, due to its biennial or short-lived perennial life-cycle, clary sage is able to quickly develop a sustainable vegetative cover over a 2- or 3-year period, and to produce large amounts of biomass (Tibaldi et al., 2010; Lydakis-Simantiris et al., 2016; Grigoriadou et al., 2020). Moreover, the cultivation of aromatic crops such as clary sage, for phytostabilization purposes on TE-contaminated sites, has previously been brought forward as a possible alternative, owing to the good quality of the produced EO regarding a potential TE-contamination (Zheljazkov et al., 2008; Angelova et al., 2016). Furthermore, clary sage was previously demonstrated tolerant to TE (Chand et al., 2015; Angelova et al., 2016), although its behavior regarding TE accumulation remains unclear (Angelova et al., 2016). Such biomass can be harvested and exploited for essential oils’ (EO) production, therefore providing high-added value marketable products. In fact, EO from clary sage are valuable in food industry as well as in perfumery and cosmetics but are also known for their antimicrobial properties (Yaseen et al., 2015; Angelova et al., 2016; Zutic et al., 2016). Besides, based on recent forecasts, the global plant products and EO market is expected to consistently increase to reach more than $10 billion in 2020 and $15.6 billion in 2026 (Allied Market Research, 2020; CBI, 2020). Based on these criteria, EO production was hence the intended valorization channel for the biomass originating from the TE-contaminated site in the present work.

However, the development of the cultivated plant species can be severely affected by the high levels of bioavailable TE present in the contaminated site (Burges et al., 2018). Hence, plant growth and phytostabilization can benefit from the addition of amendments, therefore referred to as aided phytostabilization. These either mineral, organic or microbial amendments aim at enhancing plant’s health and growth or at improving TE immobilization by precipitation, complexation or root adsorption, hence reducing their mobility and bioavailability for the plant and the living organisms (Antoniadis et al., 2017; Burges et al., 2018; Gong et al., 2018). In particular, microbial inoculants such as arbuscular mycorrhizal fungi (AMF) and ectomycorrhizal fungi have previously been used in phytomanagement trials (Dagher et al., 2020b). Indeed, AMF are known to be able to establish mycorrhizal symbiosis with most of the terrestrial plants, resulting in an improved plant growth through enhanced mineral nutrient and water acquisition as well as in a mitigation of environmental stresses (Smith and Read, 2008; Ferrol et al., 2016), valuable in both plant establishment and biomass production. In addition, AMF may be beneficial regarding TE tolerance, since they were previously described as contributing to TE immobilization and reduced uptake (Gong et al., 2018; Kumpiene et al., 2019).

The cultivation of plant species is a key and determining factor, as well as the introduction of exogenous microbial species, which are susceptible to modify the structure of the bacterial communities and their diversity in soil (Wang X. et al., 2018; Dagher et al., 2019). It is believed that the distinctive microbiome around aromatic plants is highly specific and dependant on the plant species and on the related nature and abundance of secondary metabolites, present in the root exudates (Köberl et al., 2013; Adamovic et al., 2015; Misra et al., 2019). Apart from the effects of plant’s driven changes on microbial communities, it has been previously reported that mycorrhizal inoculation can also influence the microbial community structure (Lioussanne et al., 2010; Akyol et al., 2019; Monokrousos et al., 2019; Dagher et al., 2020a). Soil bacteria may obtain carbon from AMF hyphae exudates or use the hyphae themselves as substrate (Qin et al., 2016; Gui et al., 2017), creating a niche for the growth of specific bacteria (Toljander et al., 2006; Gui et al., 2017). Furthermore, some specific bacterial taxa have been reported to be highly associated with AMF as they develop and form biofilms on the surface of the fungal hyphae or colonize AMF mycelia (Iffis et al., 2014; Qin et al., 2016). In these regards, AMF inoculation is likely to result in a shift of the bacterial communities in soil and in root of the cultivated plant.

Although the plant identity can strongly shape the bacterial communities’ structure, the combined effects of perennial clary sage inoculated with AMF under TE-contaminated conditions on bacterial diversity and communities’ structure, remain unexplored. Thus, the present work aims at (i) characterizing the bacterial richness and communities’ structure in an aged-TE agricultural contaminated soil, (ii) determining changes of both the rhizosphere soil and root’s communities over two growing seasons, and (iii) evaluating the impact of the AMF inoculation on the bacterial richness and communities’ structure overtime.

## Materials and Methods

### Experimental Design

#### Experimental Site

This study was conducted *in situ* on an aged TE-contaminated site (50°25′55.5″ N, 3°02′25.5″ E; altitude 23 m) in the north of France. A warm temperate humid climate (Csb – Köppen-Geiger classification) with cool summers characterizes the climate. The mean annual temperature is 12°C, with mean monthly temperatures ranging from 3.6°C in January to 20.3°C in July. Annual precipitation is 742.5 mm, almost equally distributed over the year (Infoclimat, 2020). The site is a former agricultural field of about 1.3 ha, located approximately 600 m away from a former lead smelter, Metaleurop Nord, whose activity during more than a century led to a topsoil (0–30 cm) contamination by TE, resulting mostly from atmospheric deposition (Sterckeman et al., 2002; Labidi et al., 2015). In particular, high amounts of Cd, Pb and Zn (7, 394, and 443 ppm, respectively) were recorded, with concentrations approximately 17-, 11-, and 6-fold higher, respectively, than those reported in regional background levels for agricultural soils (Sterckeman et al., 2007). Briefly, the soil is a silt loam, with a slightly alkaline (7.86) water pH. The main characteristics have been evaluated from 19 soil samples collected before sowing and are as follows: soil organic matter, 29.0 ± 4.0 g.kg^–1^; total N, 1.62 ± 0.24 g.kg^–1^; C/N ratio, 10.5; available (water extractable) P (Joret-Herbert), 0.17 ± 0.02 g.kg^–1^; available K, 0.26 ± 0 g kg^–1^; available Mg, 0.21 ± 0.02 g.kg^–1^; available Ca, 6.0 ± 0.5 g.kg^–1^; available Na, 0.03 ± 0 g.kg^–1^.

Clary sage (*S. sclarea L.*), a biennial or short-lived perennial aromatic plant species, has been sown in late April 2017 (300 000 seeds per hectare) and cultivated for 2 years in the contaminated site. Seeds were purchased from the French research institute ITEIPMAI, providing certified seeds.

The experimental design included two conditions [non-inoculated (NI) and inoculated with the mycorrhizal inoculum (I)] and a time monitoring (initial state of the plot, unplanted – state at first sage harvest, year 1 – state at second sage harvest, year 2), with five replicates each. A commercial mycorrhizal inoculum (AGTIV^®^ Specialty Crops, Premier Tech, QC, Canada) containing the AMF *Rhizophagus irregularis* isolate DAOM 197198 in a powder formulation (12000 viable spores per gram of product, 125 g/ha) was kindly provided by Premier Tech. It has been introduced during sowing, mixed with sage seeds.

#### Soil and Sage Root Sampling

In April 2017, five soil samples have been randomly collected from the surface horizon (0–30 cm) with a manual auger on the experimental site, to assess the site’s initial characteristics. Two other sampling campaigns have been done in August 2017, to assess the state at first sage harvest (after 21 weeks of cultivation) and in July 2018 (after 66 weeks of cultivation), to assess the state at the second sage harvest, totalizing 10 soil samples each. The study was conducted in a completely randomized experimental design and all the sampling points have been geo-referenced. The three closest growing sage plants for each sampling point were collected with their root systems. All samples were collected over a half-day period and transported within 2 h. After sampling, sage roots were vigorously shaken to remove slightly attached soil. Rhizospheric soil was then collected by means of a careful clean-up, using sterile brush and spatula tip (Sun et al., 2018; Yi et al., 2019). Sage roots were finally rinsed three more times with sterilized water to get rid of the potentially remaining soil particles. Soil and sage roots were frozen and stored at −20°C for further use.

#### Plant Growth

As previously described, sage above-ground parts have been sampled after 21 and 66 weeks of cultivation, to characterize biomass development. For each condition, fifteen plants were used as biological replicates. They were placed in an oven (72 h – 80°C), to determine their dry weights.

#### Arbuscular Mycorrhizal Root Colonization

Fresh roots were stained using Trypan blue (0.5 g.L^–1^), according to the method described by Mustafa et al. (2016) and root colonization was estimated by microscopic observation (Nikon Eclipse E600, ×100 magnification) using the grid line intersect method (McGonigle et al., 1990). In that regard, root fragments of 1 cm from three replicates per sampling point (45 fragments in total) were observed and 3 intersections per root fragment were examined. In total, more than 600 observations per condition were analyzed to assess total mycorrhizal colonization.

### DNA Extraction

#### From Soil

Genomic DNA was extracted in triplicates directly from 250 mg of soil using Nucleospin Soil^®^ kit (Macherey-Nagel, Düren, Germany), according to the manufacturer’s instructions. The quality of the extracted DNA was verified using 1% (w/v) agarose gels. Quantification of extracted DNA was carried out on a SpectraMax^®^ iD3 spectrophotometer (Molecular Devices LLC, Sunnyvale, CA, United States). The concentration of all samples was determined and DNA extracts were diluted to 25 ng.μL^–1^ for further analyses. The extracted DNA was stored at −20°C until use.

#### From Sage Roots

The fine roots were kept in Eppendorf tubes and frozen at −20°C. Genomic DNA from *S. sclarea* roots was extracted in triplicate using a method adapted from Abu-Romman (2011) and Aleksić et al. (2012). Briefly, sage roots were beforehand frozen in liquid nitrogen (−196°C) in a sterilized mortar and ground into fine powder. 500 mg of the ground roots were then subject to Cetyltrimethylammonium bromide (CTAB – 1.4 M NaCl, 100 mM Tris-HCl pH 8.0, 20 mM EDTA pH 8.0, 2% CTAB), Polyvinylpyrrolidone (PVP 1% w/v), β-Mercaptoethanol (5% v/v) and activated charcoal (0.5% w/v) extraction (30 min – 55°C). After this incubation period, a centrifugation step was carried out (10 min – 16000 *g*) and the lysate extraction was then performed using two successive steps with chlorophorm:isoamylalcohol (24:1). DNA precipitation then occurred in the presence of isopropanol (1 h incubation – 25°C), followed by another centrifugation (10 min – 700 *g*). The DNA pellet was next washed three times in a row by addition of ice-cold ethanol (70%) and centrifuged (10 min – 900 *g*), before air drying at room temperature (approximately 1 h 30 – 20°C). Finally, DNA pellet was dissolved in 50 μL of TE buffer (10 mM Tris-HCl, pH 8.0; 1.0 mM EDTA, pH 8.0). The quality of the extracted DNA was verified using 1% (w/v) agarose gels. DNA extracts’ quantification was carried out on a SpectraMax^®^ iD3 device (Molecular Devices LLC, Sunnyvale, CA, United States). The concentration of all samples was determined and DNA extracts were diluted to 25 ng.μL^–1^ for further analyses. The extracted DNA was stored at −20°C until use.

### Polymerase Chain Reaction and Sequencing

#### 16S rRNA Bacteria Gene

The V3–V4 hypervariable regions of the 16S rRNA gene were amplified from genomic DNA using a PCR thermal cycler (Agilent Surecycler 8800) with CS1 and CS2 barcoded degenerated primers as follows: forward primer: **CS1**_341_F **ACACTGACGACATGGTTCTACA**CCTACGGGNGGCWGCA G, reverse primer: **CS2**_805_R **TACGGTAGCAGAGACTTG GTCTCT**GACTACCAGGGTATCTAATC, with an expected length of about 400 bp (Herlemann et al., 2011). CS1 and CS2 tags are highlighted in bold.

Triplicate reactions per DNA sample were performed. The reaction mixtures (25 μL) contained 5 μL of Q5 (5×) reaction buffer and 0.25 μL (2 U μL^–1^) of Q5^®^ High-Fidelity DNA Polymerase (New England Biolabs France, Évry, France), 0.8 μL of each primer (0.4 μM), 1 μL of dNTPs (0.2 mM), 1 μL of DMSO, 1 μL of Bovine Serum Albumin (BSA – 100 μg/mL) and 1 ng of DNA template. The PCR conditions were as follows: initial denaturation at 95°C for 3 min followed by thirty-five cycles of 95°C for 30 s, 56°C for 30 s, and 72°C for 50 s, and a final elongation step at 72°C for 5 min.

#### Illumina MiSeq Sequencing

The triplicates for each PCR product (*n* = 25 for soil; *n* = 20 for roots) were pooled together and sent for sequencing at the Genome Quebec Innovation Centre (Montreal, QC, Canada), using Illumina MiSeq paired-end 2 bp × 300 bp.

### Data Analysis – Bioinformatic Processing

Bioinformatic processing and statistical analyses were performed using R 3.6.1 (R Core Team, 2019). A sequencing pipeline, namely DADA2 (v. 1.12), was used to process the data and to infer amplicon sequence variants (ASV), following the described procedure^1^. Briefly, DADA2 is an open-source program implemented in R package (Callahan et al., 2016), allowing to trim and filter forward and reverse reads separately, according to quality thresholds (removal of primers and low-quality nucleotides). Error rates were estimated using the error model algorithm of DADA2 for each sequencing dataset separately and reads were then deduplicated and ASVs were inferred. These ASVs were merged at the end of the pipeline, after a denoising step carried out on the forward and reverse reads independently and before a final step of chimera removal. One should note that the bioinformatic analysis was run separately for each dataset, namely “bacteria-root” and “bacteria-soil,” as these were separately sequenced and therefore a specific error model for each dataset was calculated.

Taxonomic assignment was performed through the DADA2 pipeline, using the Ribosomal Database Project (RDP) naïve Bayesian classifier (Wang et al., 2007). The Silva v132 database formatted for DADA2 (Callahan, 2018) was used to assign bacterial taxa from Kingdom to genus (minimum bootstrap 80). Rarefaction curves were visualized with the “rarecurve” function in the Vegan R package.

### Nucleotide Sequence Accession Numbers

The 16S rRNA gene sequences of the whole dataset have been deposited in NCBI Sequence Read Archive (SRA) database and can be found under accession number PRJNA648983.

### Statistical Analysis

Shapiro and Bartlett tests were used prior any statistical analysis, so as to verify the normality and the homoscedasticity of the data, respectively. If both these conditions were verified, ANOVA analysis was carried out. Otherwise, Kruskal–Wallis non-parametric test (“kruskal.test” function in R) was used. All statistical tests were considered significant with α = 0.05. Permutational multivariate analyses of variance (PERMANOVA), based on Bray-Curtis dissimilarity was run with 1000 permutations using “adonis” function and the numbers of shared ASVs between the experimental conditions were highlighted using Venn diagrams, with the help of Vegan package in R. Correlation (based on Pearson’s correlation matrix) and abundance heatmaps were plotted using “heatmap2” function from gplots package. Richness (Chao1) and diversity (Shannon and Inverse Simpson) indexes were calculated using Phyloseq package. The Chao richness estimator is a non-parametric method estimating the number of species in a community by considering rare species (singletons and doubletons – Kim et al., 2017). Both Shannon and Inv. Simpson indexes are diversity measurements. The biological diversity is widely explained as follows: “Biological diversity means the variability among living organisms from the ecological complexes of which organisms are part, and it is defined as species richness and relative species abundance in space and time” (Kim et al., 2017). Alpha diversity is assessed locally, within a specific ecosystem or area (Thukral, 2017). In that regard, both indexes are measurements of alpha diversity, based on relative abundances, but Shannon index gives a greater weight to species richness, while Simpson index gives greater prominence to species evenness (Shannon, 1948; Simpson, 1949; Kim et al., 2017).

## Results

### Descriptive Results of the Bioinformatic Outcomes

A total of 1 794 423 and 1 332 221 bacterial raw MiSeq reads were obtained for soil and root biotopes, respectively, from 25 soil samples and 20 root samples. After quality filtering and chimera removal, 21 770 and 25 415 bacterial sequences were retained for further analyses on community composition in soil and root biotopes, respectively. Bioinformatic analysis details are shown in Supplementary Table 1.

Rarefaction curves for each sample tend to reach saturation at approximately 10,000 reads (Supplementary Figure 1). As such, the rarefaction curves suggest that the depth of sequencing efforts was adequate and that most of the bacterial diversity either associated with the roots or in the rhizosphere of clary sage has been recorded. In addition, rarefaction curves reach the plateau at the same magnitude for both root and rhizospheric soil samples.

### Sage Growth and Mycorrhizal Root Colonization

In non-inoculated (NI) treatment, mycorrhizal colonization rates did not change between year 1 and year 2. They ranged between 4 and 5% (Table 1). However, in inoculated (I) treatment, a significant increase in the mycorrhizal colonization rates was observed during the second growing season, reaching 16% (*P* < 0.001). In addition, during this second season, the mycorrhizal inoculation has shown a significant increase of root colonization (69%) compared to non-inoculated plants. Regarding shoot dry weights, they increased by 50% between year 1 and year 2. They varied from 30 to 68 g.plant^–1^ and from 34 to 72 g.plant^–1^ [*F*_(__3__)_ = 7.031; *P* < 0.001], for non-inoculated and inoculated plants, respectively. No significant differences were observed for plant’s dry weights between NI and I conditions, whatever the year [*F*_(__3__)_ = 0.476; *P* = 0.701].

**TABLE 1:** Sage dry weight and root total mycorrhizal colonization in response to NI and I conditions.

	Year 1		Year 2	
	NI	I	NI	I
Total mycorrhizal rate (%)	4 ± 4^a^	1 ± 1^a^	5 ± 2^a^	16 ± 5^b^
Dry weight (g.plant^–1^)	30 ± 9^a’^	34 ± 10^a’^	68 ± 16^b’^	72 ± 31^b’^

*Values are means ± SD (*n* = 5). Different lowercase letters indicate significant differences between the four tested experimental conditions and are displayed without and with apostrophe for total mycorrhizal rates and dry weight, respectively, as the statistical significance has been evaluated separately (ANOVA – α = 0.05). NI, non-inoculated; I, inoculated.*

### Bacterial α-Diversity in Root and Soil Biotopes

The bacterial α-diversity was calculated separately for each experimental condition, *i.e.*, culture duration (initial state, year 1 and year 2) and mycorrhizal inoculation (NI or I), and accordingly for root and soil biotopes. According to the Chao1 index, the bacterial species richness in soil decreased for year 2, especially for the non-inoculated plants (Table 2). However, it significantly increased between year 1 and year 2 for bacterial communities in roots. For both root and soil biotopes, there was no significant difference between non-inoculated and inoculated treatments, whatever the year.

**TABLE 2:** Richness and diversity indexes from the 5 studied conditions for soil bacterial communities and the 4 studied conditions for root bacterial communities.

		Soil				Roots			
	Initial state	Year 1		Year 2		Year 1		Year 2	
		NI	I	NI	I	NI	I	NI	I
Chao1	177237^a^	184730^a^	178163^a^	757268^bc^	131533^ab^	44348^c^	65970^bc^	90050^b^	90881^b^
Shannon	6.70,05^ab^	6.70.08^a^	6.70.09^a^	5.01.24^b^	6.00.14^ab^	3.50.42^c^	4.40.68^b^	4.60.5^b^	4.70.33^b^
Inv. Simpson	335.041.0^a^	346.383.3^a^	387.374.4^a^	75.944.1^b^	117.630.9^ab^	11.13.2^c^	36.826.5^bc^	19.28.9^c^	17.03.3^c^

*Data are means ± SD (*n* = 5) for each condition. Significant differences between experimental conditions in both soil and root biotopes are indicated by different letters. NI, non-inoculated; I, inoculated.*

No significant difference was observed for Shannon and Inverted Simpson diversity indexes for soil bacteria (*P* = 0.6); they were similar between the soil before plantation (initial state) and year 1, but they decreased in year 2 with a significant reduction for non-inoculated plants (*P* = 0.002). However, there was no significant difference between non-inoculated and inoculated treatments, regardless the year of sampling and the diversity index.

In root samples, bacterial α-diversity was significantly different between year 1 and year 2 (Shannon index only) for non-inoculated plants (*P* = 0.008). In addition, during year 1, diversity was significantly increased in inoculated plants in comparison with the non-inoculated ones (*P* = 0.03), and that for both Shannon and Inv. Simpson indexes. However, no significant difference was found between inoculated and non-inoculated treatments for year 2 (*P* = 0.9). In comparison with the soil biotope, all the indicators in roots displayed significantly lower (*P* = 0.001) values, resulting in lower bacterial richness and diversity.

### Evaluation of the Potential Effects of Sampling Time and Mycorrhizal Inoculation on the Bacterial Community Structure

**FIGURE 1:**
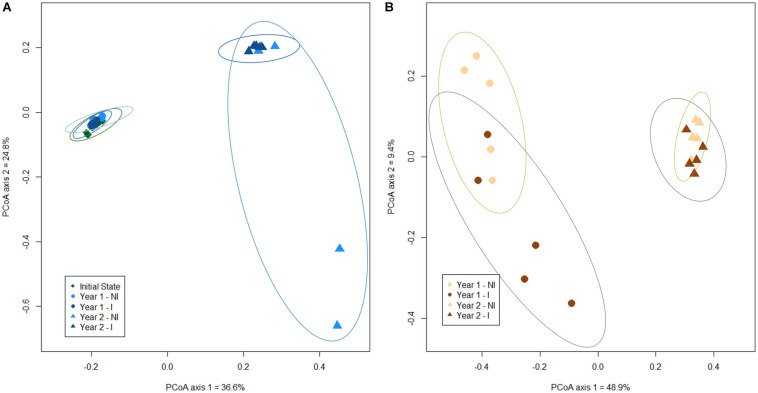
Principal coordinate analysis (scaling 1) based on Bray-Curtis dissimilarity for soil **(A)** and root **(B)** bacterial ASVs in the different experimental conditions. Confidence area of ellipses = 0.95. NI, non-inoculated; I, inoculated.

To investigate the influence of the sampling time (before plantation, year 1 and year 2) and mycorrhizal inoculation (NI or I) on root and rhizospheric soil bacterial communities, a principal coordinate analysis (PCoA) ordination was conducted. Our results have shown a net clustering of bacterial communities, in both rhizospheric soil and root biotopes, according to the sampling time. Of the total variance in the dataset, the first two principal components together explained 61.4% of the total soil bacterial communities and 58.3% of the total root bacterial communities. For soil bacterial communities, a grouping between soil before plantation and year 1 communities was operated, distinct from year 2 bacterial community clustered together, highlighting a time effect (Figure 1A). However, there was no distinction between soil bacterial communities in non-inoculated or inoculated treatments, regardless of the sampling time. Similarly, bacterial communities in roots from year 1 and year 2 were clustered separately, depicting a time effect, but no distinction was made between non-inoculated and inoculated treatments, whatever the year (Figure 1B). However, one should note a greater heterogeneity in soil bacterial communities for year 2, with the inoculated treatment displaying a higher between-sample variation than the non-inoculated one. The opposite trend is visible for root bacterial communities, with a higher heterogeneity for the inoculated treatment during year 1. In addition, the results of the PERMANOVA analysis indicated that the composition of the bacterial communities was significantly different overtime (*P* < 0.001), in both rhizospheric soil and roots, but that inoculation’s effect was not significant in both biotopes (*P* > 0.05 – Supplementary Figure 2).

**FIGURE 2:**
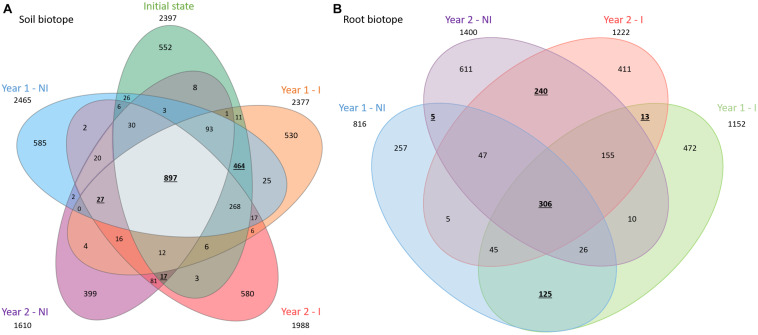
Venn diagrams showing the overlap of the bacterial communities overtime, with NI condition on one side (left side) and I condition on the other (right side) and for soil **(A)** and root **(B)** bacterial communities. These diagrams display the number of total (out of the circles), specific and shared ASVs. The total number of bacterial ASVs is 4691 in soil and 2728 in root. NI, non-inoculated; I, inoculated. Numbers reported in text are displayed in bold and underlined.

In total, 2397, 2465, 2377, 1610, and 1988 bacterial ASVs were obtained in the rhizospheric soil before plantation, year 1 non-inoculated or inoculated and year 2 non-inoculated or inoculated, respectively. Besides, a total of 816, 1152, 1400, and 1222 bacterial ASVs were present in the root biotopes and for year 1 non-inoculated or inoculated and year 2 non-inoculated or inoculated treatments, respectively (Figure 2). The number of shared ASVs between all treatments was 897 in the rhizospheric soil biotope and 306 in the root biotope, respectively, corresponding to 19 and 11% of the total ASVs. Among them, the soil before plantation and at the first sage harvest (year 1) shared the highest number of ASVs in soil, whatever the treatment (NI or I), representing a number of 464 shared ASVs (Figure 2A). Conversely, soil before plantation and at the second sage harvest (year 2), regardless of the treatment (NI or I), shared a number of 17 ASVs, while year 1 and year 2 only have 27 ASVs in common, depicting a net shift between year 1 and year 2. In roots, the highest number of ASVs is shared between I and NI conditions, whatever the year, representing 125 shared ASVs for year 1 and 240 for year 2 (Figure 2B). On the contrary, year 1 and year 2 NI treatments only shared 5 ASVs, while year 1 and year 2 I treatments have 13 ASVs in common, depicting a change in time of the bacterial community composition. In addition, the percentage of ASVs specific to each condition is comparable and ranged from 22 to 29% for soil bacterial communities and from 31 to 44% for root bacterial communities.

### Changes Induced at the Phylogenetic Level by the Cultivation of Clary Sage and Mycorrhizal Inoculation on the Bacterial Community’s Composition

**FIGURE 3:**
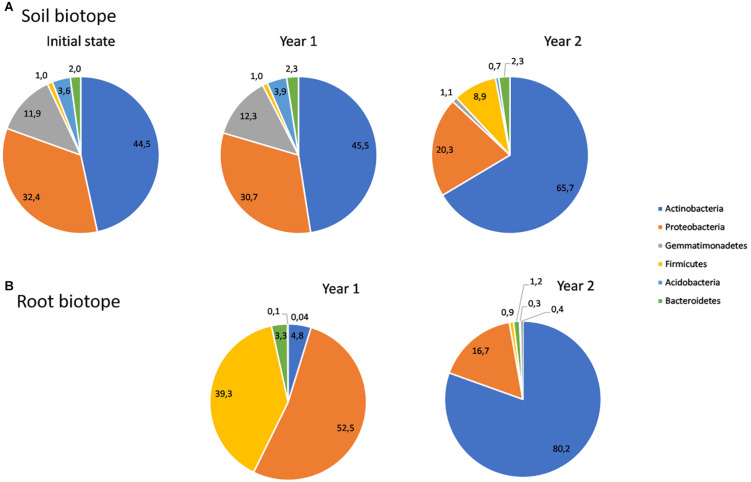
Relative abundances of bacteria belonging to the most abundant phyla in soil **(A)** and root **(B)** biotopes.

To assess the community composition, each bacterial ASV was assigned to phyla, classes, families and genera. Soil bacterial ASVs were assigned to 24 phyla, while root ASVs were assigned to 20 phyla. *Actinobacteria* are the most represented one in soil, whatever the treatment. In particular, it represented 45% of bacterial ASVs for the soil before plantation (initial state) and year 1, increasing to 66% for year 2, regardless of the treatment (Figure 3A). *Proteobacteria, Gemmatimonadetes*, *Firmicutes, Bacteroidetes*, and *Acidobacteria* are also among the most represented phyla in soil, as combined together, alongside with *Actinobacteria*, they represent nearly 95% of the soil bacterial communities. The *Firmicutes* phylum follows the same trend as *Actinobacteria* and its proportion increased in year 2. *Bacteroidetes* have shown a stable trend overtime, while an opposite trend was observed for the other three phyla, whose proportion consistently decreased in year 2. Regarding root bacterial communities, the same six phyla have been found as the most represented in the communities, containing more than 99% of the ASVs. In comparison to the soil biotope, *Actinobacteria* were poorly represented in year 1, accounting for less than 10% of the ASVs. However, they significantly increased to 80% in year 2. *Proteobacteria* and *Firmicutes* have undergone the opposite trend between year 1 and year 2, decreasing from 53 to 17% and 39 to 1%, respectively (Figure 3B).

**FIGURE 4:**
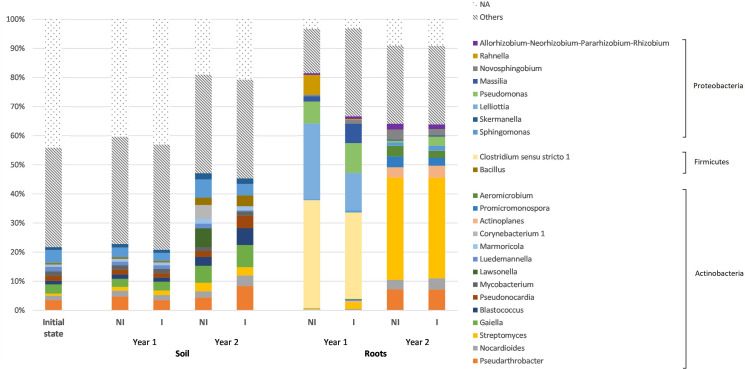
Relative abundances of the 25 most represented bacterial genera, according to the different experimental conditions for both root and soil biotopes. The genera have been grouped under their corresponding phyla and genera with a relative abundance lower than 0.5% have been gathered in the “others” group. NI, non-inoculated; I, inoculated; NA, unassigned.

At the genus level, the dominant genera among soil bacteria included *Sphingomonas*, *Pseudarthrobacter*, *Nocardioides*, *Streptomyces*, *Gaiella*, *Blastococcus*, and *Pseudonocardia* (Figure 4). In particular, *Sphingomonas*, *Pseudarthrobacter*, *Streptomyces*, and *Gaiella* were the most dominant genera. However, a high number of ASVs (approximately 40%) was not assigned to any genus. In addition, one should note that the proportions between the most represented bacterial genera in soil, under the two experimental conditions (NI or I) and the three monitoring times (soil before plantation, year 1 and year 2) were not significantly different (statistical analysis results provided in Supplementary Table 2).

Regarding root bacterial communities, at the genus level, *Clostridium sensu stricto* 1, *Lelliottia*, *Pseudomonas*, *Rahnella*, *Streptomyces*, *Pseudarthrobacter*, and *Nocardioides* are the most dominant genera (Figure 4). However, the proportion of dominant bacterial genera was significantly different (*P* < 0.05), especially between the two monitoring times (year 1 and year 2) (Supplementary Table 2). In particular, *Clostridium sensu stricto* 1, *Lelliottia* and *Pseudomonas* that were extensively represented in year 1, with respective proportions of 37 and 30%; 26 and 13%; and 8 and 10% (in NI and I treatments, respectively), decreased in year 2 to, respectively, 0.03 and 0.04%; 0.02 and 0.01%; and 0.6 and 3.2% (for NI and I treatments, respectively). Conversely, *Streptomyces*, *Pseudarthrobacter*, and *Nocardioides* were scarcely represented in year 1 (less than 2% for each genus in any treatment), whereas their relative abundances have significantly increased in year 2, with respective proportions of 35 and 35%; 7 and 7%; and 3 and 4% (for NI and I treatments, respectively). In addition, there is no significant difference between non-inoculated and inoculated treatments, whatever the biotope and the monitoring time (Supplementary Table 2).

### Correlations Between the Most Abundant ASVs and Sage Dry Weight and Root Mycorrhization

**FIGURE 5:**
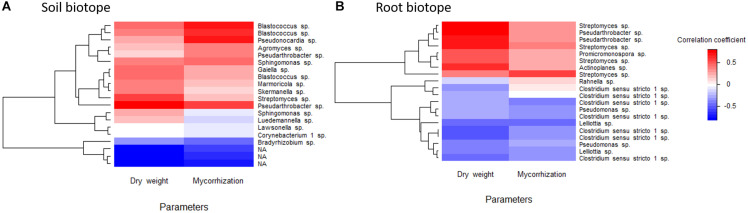
Heatmap based on the correlation coefficients between clary sage dry weight and root mycorrhization and the 20 most represented bacterial ASVs in soil **(A)** and root **(B)** biotopes, belonging, respectively, to 13 and 8 bacterial genera. NA, unassigned.

A heatmap based on Pearson correlation coefficients between the 20 most abundant ASVs and sage dry weight and root mycorrhization as explaining parameters was drawn, for rhizospheric soil and root biotopes (Figure 5). These correlation coefficients ranged from −0.68 to 0.69 and from −0.51 to 0.81 for the rhizospheric soil and root biotopes, respectively. *Pseudarthrobacter* and *Streptomyces* were the genera presenting the highest correlation coefficients regarding sage dry weight, while *Blastococcus* and *Pseudonocardia* were the strongest positively correlated ones in soil regarding root mycorrhization. Similarly in roots, *Streptomyces*, *Pseudarthrobacter*, and *Actinoplanes* are strongly and positively correlated with sage dry weight, while *Streptomyces* also displayed the highest and positive correlation coefficient regarding sage root mycorrhization. Conversely, *Corynebacterium* 1 or *Lawsonella* in the soil biotope are not correlated to any of the parameters, while in the root biotope, *Clostridium sensu stricto* 1 and *Lelliottia* are negatively correlated to both sage dry weight and root mycorrhization.

Bacterial ASVs that were assigned a genus were further investigated to establish whether these genera contain plant growth promoting-rhizobacteria (PGPR) species or not, based on a literature survey. Relative abundances of ASVs that were assigned to genus level and those that comprise known PGPR species are shown in Table 3. In soil before plantation and at year 1, these ASVs represented about 40% of the soil bacterial communities. For year 2 and for non-inoculated plants, this proportion remained unchanged, whereas it increased to 64% for inoculated plants. In roots, PGPR-containing genera represented about 25% of the bacterial communities in year 1, increasing to 76% for year 2, regardless of inoculation treatment (Table 3). Nitrogen-fixing genera, such as *Rhizobium* or *Bradyrhizobium* only accounted for a small proportion of the PGPR-containing genera, whatever the treatment, the year and the biotope.

**TABLE 3:** Relative abundances of the 100 most abundant bacterial ASVs belonging to 24 genera, in which plant growth promoting-rhizobacteria (PGPR) have previously been identified.

		Soil				Roots			
		Year 1		Year 2		Year 1		Year 2	
Genus	Initial state	NI	I	NI	I	NI	I	NI	I
*Agromyces*	1.50.3cd	0.020.02e	0.10.1de	0.40.1de	0.40.2de	1.70.1cd	1.60.3cd	0.71.3cde	1.90.4cd
*Amycolatopsis*	nd	0.070.19*e*	0.30.5de	2.22.3cd	1.31.3cd	nd	nd	nd	nd
*Bacillus*	0.50.1de	nd	nd	nd	nd	0.60.3de	0.60.3de	1.71.4cd	2.21.0cd
*Blastococcus*	3.21.0c	nd	nd	nd	nd	4.00.7c	3.90.8c	4.76.7c	12.03.2bc
*Bradyrhizobium*	4.01.2c	0.20.1de	0.60.8de	0.40.2de	0.91.0cde	4.20.7c	3.40.9c	1.11.2cd	0.10.08de
*Caulobacter*	nd	0.10.09de	0.30.5de	0.10.1de	0.60.5de	nd	nd	nd	nd
*Cellulomonas*	1.00.5cd	0.060.08e	0.30.2de	0.30.3de	0.030.08e	1.10.3cd	1.10.2cd	1.23.2cd	2.40.7c
*Flavobacterium*	nd	0.40.3de	2.21.5cd	nd	nd	nd	nd	nd	nd
*Kribbella*	3.01.1c	0.040.04	0.20.1de	0.70.3cde	1.10.3cd	3.31.4c	3.10.8c	0.30.8	1.10.2cd
*Massilia*	nd	1.30.2cd	7.22.8c	0.10.03de	0.40.1de	nd	nd	nd	nd
*Microvirga*	0.90.3cde	nd	nd	nd	nd	1.00.1cd	0.70.1cde	0.040.1e	0.10.1de
*Nocardioides*	1.40.6cd	0.050.02e	0.20.1de	1.60.2cd	2.40.4cd	1.90.5c	2.10.5c	1.55.0c	3.51.7c
*Novosphingobium*	nd	0.30.2de	0.70.3de	4.02.1c	2.20.7cd	nd	nd	nd	nd
*Pantoea*	nd	0.90.9cd	0.20.2de	0.030.06e	nd	nd	nd	nd	nd
*Pseudarthrobacter*	11.24.3bc	0.080.05e	0.30.1de	10.82.2c	10.43.0c	14.95.0bc	11.13.3bc	7.713.2c	20.65.6b
*Pseudomonas*	nd	6.40.8c	11.11.5bc	0.40.05de	4.61.2c	nd	nd	nd	nd
*Pseudonocardia*	4.40.5c	nd	nd	nd	nd	4.30.4c	4.00.7c	2.73.5c	8.62.9c
*Rahnella*	nd	7.83.6c	0.40.2de	0.0060.005e	nd	nd	nd	nd	nd
*Rhizobium*	nd	0.30.2de	0.80.5cde	2.00.8cd	1.70.4cd	nd	nd	nd	nd
*Serratia*	nd	1.71.1cd	0.50.3de	0.020.03e	nd	nd	nd	nd	nd
*Shinella*	nd	0.070.07e	0.30.4de	0.20.1de	0.20.2de	nd	nd	nd	nd
*Sphingomonas*	8.63.1c	nd	nd	0.30.3de	0.81.0de	6.01.9c	5.41.2c	9.010.2c	7.511.0c
*Streptococcus*	nd	nd	nd	nd	nd	nd	nd	2.31.8cd	nd
*Streptomyces*	0.40.06de	0.40.03de	3.20.3c	52.51.5a	50.41.0a	1.30.7cd	1.20.5cd	3.63.6c	3.91.8c

*Values are means ± SD (*n* = 5). Significant differences (Kruskal–Wallis non-parametric test, α = 0.05) between conditions are indicated by different letters. NI, non-inoculated; I, inoculated; nd, not-detected.*

## Discussion

Clary sage is one of the most important aromatic plants cultivated worldwide for its EO in perfumery and cosmetics (Tibaldi et al., 2010; Angelova et al., 2016). There is a growing demand in *S. sclarea* EO and an abundant flowering leading to elevated EO yields are sought (Zutic et al., 2016). If clary sage was able to grow consistently on TE-contaminated soils, and to be established in such conditions, it could represent a valuable economic alternative for the reconversion of these marginal lands in a context of a growing arable land’s demand. The present study focused on the rhizosphere bacterial community, given its contribution in ecosystem functioning and services (Delgado-Baquerizo et al., 2016; Foulon et al., 2016b; Dagher et al., 2019). It is in particular well known that the rhizosphere microbial communities are an important factor influencing plant growth and subsequently biomass and EO production (Wang X. et al., 2018; Akyol et al., 2019; Berlanas et al., 2019).

As far as we know, clary sage microbiota, however, remains unexplored and on both polluted and healthy soils, even though the microbiota of some other aromatic plant species, such as *Ocimum basilicum* L., *Mentha x piperita*, *Anethum graveolens* L., *Calendula officinalis* L., *Mentha arvensis* L., *Artemisia annua* L., and *Panax ginseng* Meyer have previously been examined in agricultural soils (Adamovic et al., 2015; Misra et al., 2019; Wei et al., 2020). Hence, the current study represents the first investigation on the effects of revegetation using clary sage, on both rhizospheric soil and roots’ bacterial communities under TE-contaminated conditions. Furthermore, the potential influence of a biological amendment’s introduction (AMF inoculum) on bacterial communities’ structure, richness and diversity and their evolution over successive sage cultivation years has been studied.

### Dominance of *Actinobacteria* and *Proteobacteria* Phyla in the Rhizosphere Microbiota of the Aged TE-Contaminated Soil

*Actinobacteria*, *Proteobacteria*, *Gemmatimonadetes*, *Firmicutes*, *Bacteroidetes*, and *Acidobacteria* were found to be the most represented phyla in the aged TE-contaminated soil before plantation and during the two successive growing seasons of clary sage. The prevailing phyla identified in the sage roots were found to be similar to those detected in the soil. These findings are in accordance with previous studies carried out in agricultural and TE-contaminated soils, involving endemic plant species such as *Silene vulgaris* (Moench) Garcke, *Bahia xylopoda* Greenm., or *Viguiera linearis* Cav. (Navarro-Noya et al., 2010; Pereira et al., 2015; Ahmad et al., 2016; Pires et al., 2017; Pacwa-Płociniczak et al., 2018). However, variations were observed between our results and previously published studies, in terms of relative abundances between the most represented phyla. One should keep in mind that changes observed between TE-contaminated sites are highly related to several factors, including the climatic conditions, the TE-contamination level, the cultivation practices as well as the soil physico-chemical characteristics, especially pH variations (Wang et al., 2019). As such, *Acidobacteria* and *Proteobacteria* have been demonstrated to decrease with increasing pH (Wang et al., 2019). In the present study, however, the soil pH was stable overtime under the different experimental conditions and was relatively high in comparison with acidic mine tailings (Navarro-Noya et al., 2010; Pereira et al., 2015). This could be responsible for the relatively low abundance of *Acidobacteria* and *Proteobacteria* observed in our conditions. As soil microbial community structure is driven by dominant sources of bioavailable C, plant species is then another determinant factor (Köberl et al., 2013; Monokrousos et al., 2019; Wang et al., 2019). In particular, plants are able to secrete specific root exudates and to modulate their nature (sugars, sterols, amino and organic acids), hence shaping the microbial communities’ structure accordingly in the rhizosphere (Wang X. et al., 2018; Misra et al., 2019). Diversity indexes obtained in the present study are similar or higher in comparison with previous reports on revegetated or agricultural TE-contaminated soils (Foulon et al., 2016a; Xie et al., 2016; Tipayno et al., 2018). However, the comparison of alpha diversity between experimental sites is tricky, as it highly depends on cultivation practices amongst several parameters (soil type and physico-chemical properties, climatic conditions and plant species) (Wang X. et al., 2018, Wang et al., 2019).

Interestingly, among the identified phyla, *Actinobacteria, Proteobacteria*, *Firmicutes*, and *Bacteroidetes* were previously demonstrated to possess TE-tolerance genes, coding for proteins involved in efflux and sequestration phenomena of TE ions, such as Cu^2+^ or Cd^2+^/Zn^2+^-exporting ATPases, putative copper resistance protein D or copper transport protein B and in the mitigation of oxidative stress induced by the presence of TE, such as superoxide dismutase, alkyl hyperoxide reductase or mycothiol reductase (Chen et al., 2018; Cui et al., 2018; Hemmat-Jou et al., 2018). Besides, *Actinobacteria* and *Proteobacteria*, which are the two prevailing bacteria phyla in the present work whatever the biotope, were previously brought forward as reducing TE accumulation in corn. Notably, it is believed that both bacterial phyla could alter the bioavailability of heavy metals via redox reactions, precipitation, as well as sorption and desorption reactions (Cui et al., 2018). More specifically, *Bacillus*, *Pseudomonas* and *Arthrobacter*, respectively, belonging to *Firmicutes*, *Proteobacteria*, and *Actinobacteria* phyla, are among the most widely spread and frequently listed bacterial genera in TE-contaminated soils, and at the same time display a high TE tolerance (Pires et al., 2017; Chellaiah, 2018; Chen et al., 2018; Cui et al., 2018). Nonetheless, *Sphingomonas*, member of the *Proteobacteria* (Xie et al., 2010; Chen et al., 2014; Jaafar, 2019), as well as *Pseudarthrobacter* (Chen et al., 2014; Fekih et al., 2018; Park et al., 2019), *Nocardioides* (Bagade et al., 2016; Cui et al., 2018), and *Streptomyces* (Álvarez et al., 2013; Cui et al., 2018; Durand et al., 2018; Lasudee et al., 2018; Timková et al., 2018; Álvarez-López et al., 2020), belonging to the *Actinobacteria*, were also highlighted for displaying TE-tolerance and even studied for potential remediation applications. Indeed, these bacterial genera are known to accumulate Cd, Ni, Cr or Pb from TE-contaminated soils and to induce changes in the oxidation state of the TE (Pires et al., 2017; Chellaiah, 2018; Timková et al., 2018).

### Significant Effect of Sampling Time on the Bacterial Community Structures in the Root and Soil Biotopes

Regarding richness and diversity in soil, a significant drop has been observed between the two successive years of growth, whatever the index (Chao1 estimator, Shannon and Inv. Simpson). However, it is noteworthy that an increase in both richness and diversity was observed in roots in year 2. The results from PERMANOVA and PCoA analyses have clearly indicated a year-to-year variability of the bacterial community structures in both soil and root biotopes with a high significance at *P* < *0.001*, displaying in soil a clear clustering with the soil before plantation and year 1 on one side and year 2 on the other, while in root year 1 and year 2 were clustered separately. These results fall into line with the Venn-diagrams, displaying a high number of shared ASVs between the soil before plantation and year 1 (soil biotope), as well as a drop in the total number of ASVs in soil in year 2, and a scarcer number of shared ASVs between year 1 and year 2, for both rhizospheric soil and root biotopes.

Similar results were observed in the soil at the phylum level between the soil before plantation and year 1, whereas there was a clear shift between sampling year 1 and year 2. Indeed, the *Gemmatimonadetes* and Proteobacteria phyla decreased between years 1 and 2, with respective declines of 90 and 34%, while the *Firmicutes* phylum was more represented in year 2, increasing of about 90% in comparison with year 1. One should note that *Actinobacteria* was the prevailing phylum at any of the sampling times, in addition displaying a significant increase of about 30% between years 1 and 2. A major shift has also been observed in roots between year 1 and year 2, with *Firmicutes* and *Proteobacteria* representing more than 90% of the ASVs in year 1, while *Actinobacteria* was the prevailing phyla in year 2, accounting for 80% of the ASVs. At the genus level, a major shift was also observed especially in the root biotope between year 1 and year 2, with the prevalence of *Clostridium sensu stricto* 1, *Lelliottia* and *Pseudomonas* for year 1, respectively, belonging to *Firmicutes* and *Proteobacteria* phyla, while year 2 was clearly dominated by *Pseudarthrobacter, Nocardioides*, and *Streptomyces*, all belonging to the *Actinobacteria.*

Thus, the shift observed with the culture duration in both biotopes but particularly pronounced in the case of roots, at the phylum and genus phylogenetic levels, could be explained by different factors. First, the prevalence in year 2 for both root and rhizospheric soil biotopes of *Actinobacteria*, previously demonstrated as TE-tolerant, could support the idea that the phylotypes possessing TE tolerance are susceptible to persist overtime, while sensitive ones become less frequent (Cui et al., 2018). In addition, it was previously evidenced for wheat grown in uncontaminated agricultural fields that *Actinobacteria* abundance was increasing with root age, which is possibly explained by their ability to degrade secondary cell walls containing complex molecules (Donn et al., 2015). Furthermore, environmental factors influencing root response and exudation, such as temperature or precipitation, could be responsible for this year-to-year variation (Wagner et al., 2016; Berlanas et al., 2019). In fact, it was previously demonstrated in the case of Eucalyptus or grapevine, that distinct bacterial communities were observed at different plantation ages (Berlanas et al., 2019; Qu et al., 2020). In particular, changes in exudation profiles are susceptible to occur with plant age, at distinct growth stages (Akyol et al., 2019). Similarly, in the case of aromatic plant species, such as *M. arvensis* or *A. annua*, the long-term mono-cultivation was brought forward as an explaining factor of bacterial communities’ diversity and evenness (Misra et al., 2019). Moreover, aromatic plant species like clary sage are susceptible to release secondary metabolites in soil. These metabolites, due to their potential antimicrobial nature, are likely to exert adverse effects and hence affect both richness and diversity by shaping specifically microbial communities (Köberl et al., 2013; Adamovic et al., 2015; Misra et al., 2019). A previous study on cultivated aromatic plants (*M. arvensis* and *A. annua*), demonstrated that the long-term monoculture of aromatic species could durably affect the bulk soil and rhizosphere microbial communities (Misra et al., 2019). In particular, it was suggested that menthyl acetate and menthol (monoterpenes), present in *M. arvensis*, as well as artemisinin (sesquiterpene) in *A. annua*, known for their antibacterial properties and found in the rhizospheric and bulk soil of these plant species, could greatly affect soil bacterial communities, notably through a reduction of the total soil microbial diversity (Misra et al., 2019). In the case of clary sage, linalool (terpene alcohol) and linalyl acetate (ester) have been identified as EO major compounds, and have frequently been reported for their antibacterial properties against several bacterial genera, such as *Staphylococcus, Pseudomonas, Bacillus, Listeria, Enterococcus, Mycobacterium*, or *Escherichia* (Kuzma et al., 2009; Angelova et al., 2016; Zutic et al., 2016; Blaskó et al., 2017; Aćimović et al., 2018). In that regard, the prevalence of secondary metabolites during the second year of sage growth and cultivation (Tibaldi et al., 2010) may then be another key driver (Köberl et al., 2013; Adamovic et al., 2015; Misra et al., 2019). However, there is no specific pattern regarding the previously mentioned genera in the current study. In soil, *Pseudomonas* and *Mycobacterium* were found stable during the successive cultivation years, while *Bacillus* and *Staphylococcus* were demonstrated increasing in year 2, whereas in roots, only *Pseudomonas* significantly decreased in year 2. Interestingly, our results have shown that sage was capable of growing consistently in TE-contaminated conditions, which is in accordance with previous works reporting the TE-tolerance of this aromatic plant species (Chand et al., 2015; Angelova et al., 2016).

### Mitigated Effects of AMF Inoculation in Shaping Bacterial Communities

Surprisingly, AMF inoculation, which is likely to result in a shift of the bacterial communities in soil and in root of the cultivated plants did not lead to tremendous changes in our experimental conditions (Lioussanne et al., 2010; Rodríguez-Caballero et al., 2017; Akyol et al., 2019; Monokrousos et al., 2019). No significance of AMF inoculation either in the PERMANOVA analysis or in the Venn diagram representation was observed, with a high number of shared ASVs between inoculated and non-inoculated treatments, whatever the biotope and the year of sampling. In addition, no striking difference was highlighted regarding richness and diversity indexes, between non-inoculated and inoculated treatments, whatever the cultivation year. Interestingly, the decrease of richness and diversity indexes observed in soil between successive cultivation years is less marked in the case of inoculated plants, suggesting that mycorrhizal symbiosis is likely to improve the resilience of the plant and its associated microbial communities against biotic and abiotic stresses (Bi et al., 2018). In previous studies, the mycorrhizal symbiosis was also highlighted to result in changes in the host plant metabolism and hence in the root exudation profile, further modifying and shaping the bacterial communities, in both TE-contaminated and uncontaminated conditions (Harrison, 1999; Hause and Fester, 2005; Ferrol et al., 2016; Iffis et al., 2017). In addition, the exudation of carbohydrates and citric acid, detected in AMF hyphal exudates, and their quantitative and qualitative patterns were brought forward as potential key drivers in the changes of the soil bacterial communities as well (Lioussanne et al., 2010; Iffis et al., 2016; Qin et al., 2016). However, in the present work carried out on agricultural contaminated soils, despite the presence of TE, the soil physicochemical properties were considered sufficient for low demanding crops such as clary sage (Denoroy et al., 2004; Sharma and Kumar, 2012; Abbaszadeh et al., 2017). Consequently, sage nutritional needs were probably met. Thus, even though the mycorrhizal colonization was successfully established, even displaying an increase for the inoculated treatment in year 2, it did not result in an improved plant growth in comparison with the non-inoculated plants. Hence, considering this limited contribution it may not have resulted in changes in bacterial communities in both rhizospheric soil and root biotopes or to a very limited extent. Nonetheless, as sage was effectively colonized by AMF, the monitoring of the symbiosis’ functionality through the expression of phosphate transporters genes, such as MtPT4, LePT1, or StPT3 (Paszkowski et al., 2002; Karandashov and Bucher, 2005; Voß et al., 2018), might validate this hypothesis.

### Prevalence of PGPR in Soil and Root Bacterial Communities

Although it is difficult to infer the ecological function of a bacterium based on its taxonomic assignment (Akyol et al., 2019), Pearson’s correlation analysis revealed significant and positive correlations between the most abundant ASVs in soil and root biotopes with sage dry weight and root mycorrhizal rates as explaining parameters. More specifically, *Pseudarthrobacter*, *Streptomyces*, and *Actinoplanes* genera were found strongly correlated with sage dry weight in either one or both biotopes. It was in fact previously demonstrated that the rhizosphere bacterial community could be of major importance regarding the suppression of soilborne diseases (Chen et al., 2020). In particular, *Streptomyces* spp. are known to produce a wide range of secondary metabolites with antimicrobial properties and hence considered as antagonists of both plant pathogenic or non-pathogenic fungi and bacteria (Golinska et al., 2015; Al_husnan and Alkahtani, 2016; Vurukonda et al., 2018; Al-Ansari et al., 2019). They could play an important role in shaping the bacterial communities, especially since their abundance in the present work is relatively elevated. Interestingly, our results have shown that *Streptomyces* sp. were strongly correlated to both sage dry weight and mycorrhizal rates in the root biotope, which falls in line with previous studies describing *Streptomyces* spp. as promoting the growth of numerous plant and microbial species (Foulon et al., 2016a; Iffis et al., 2016; Dias et al., 2017; Durand et al., 2018; Vurukonda et al., 2018; Wang C. et al., 2018). It hence could be considered of great interest as both PGPB and mycorrhiza helper bacteria, depending on the species and the strain. Similarly, *Pseudarthrobacter* which was found well correlated with sage biomass in the present work was recently demonstrated as a bacterial genus containing PGPB species of several plants, including other aromatic species such as *Pellaea calomelanos* Sw. (Tshishonga and Serepa-Dlamini, 2020), or *Dodonaea viscosa* Jacq., *Fagonia indica* L., *Caralluma tuberculata* N.E. Brown and *Calendula arvensis* L. (Iqrar et al., 2019). Interestingly, among the most abundant ASVs correlated with sage biomass in the roots’ biotope, two of them were assigned to *Promicromonospora* and *Actinoplanes* genera. These 2 genera were previously highlighted as increasing plant growth promotion when belonging to microbial consortia, playing a role as “activators” and enhancing the effects of other PGPB microorganisms when present in combination (El-Tarabily et al., 2010; Golinska et al., 2015). *Pseudonocardia*, which was the highest correlated ASV with sage root mycorrhization in the rhizospheric soil biotope, was previously described as a mycorrhiza helper bacteria isolated from the AMF *Funneliformis mosseae* spores (Lasudee et al., 2018). Conversely, *Clostridium* genus which was in our experimental conditions negatively correlated to both sage dry weight and root mycorrhization, is rather known for causing bacterial soft rots, especially in potato and sweet potato (da Silva and Clark, 2013; Kõiv et al., 2015). Similarly, *Lelliottia* has so far only been reported as responsible for onion bulb decay (Bonasera et al., 2014; Liu and Tang, 2016). Nevertheless, none of these two genera has previously been evidenced as a clary sage pathogen.

## Conclusion

In this study, we have investigated the effects of sampling time over a period of 2 years of growth and mycorrhizal inoculation on rhizosphere and root bacterial communities in the case of the aromatic plant, clary sage, cultivated on the aged TE-contaminated soils. *Actinobacteria* and *Proteobacteria* were found to be the predominant phyla during the 2 years of clary sage cultivation in our aged TE-contaminated soil. Altogether, our findings have demonstrated that the most important factor shaping the bacterial community in our experimental setup was the sampling time, with a significant year-to-year change in terms of bacterial α-diversity and communities’ structure in both root and rhizospheric soil biotopes. Conversely, AMF inoculation did not lead to remarkable changes in our experimental conditions. Only a less pronounced decrease in terms of bacterial richness and diversity in soil between successive cultivation years was observed in the inoculated treatment. Our results have also shown that some bacterial genera, such as *Pseudarthrobacter*, *Streptomyces* and *Actinoplanes*, were strongly and positively correlated to both sage dry weight and mycorrhizal rates in the root biotope. They hence could be considered of great interest in a phytomanagement context, since among them some bacterial species have previously been reported as both PGPR and highly tolerant to TE.

Thus, a more comprehensive study aiming at deciphering the underlying mechanisms in the shaping of the bacterial communities by clary sage and the specific interactions between the plant and the microbiota would facilitate the development of phytomanagement methods based on clary sage cultivation. In the same perspective, it may provide new insights to isolate culturable bacteria associated to clary sage grown on TE-contaminated soils and to investigate their potential as PGPR species as well as their ability to tolerate or even to mitigate TE-toxicity. Nonetheless, a similar approach targeting the fungal community is also relevant and could help gaining more insights into the complex interactions within the rhizosphere microbiota. In fact, both fungal and bacterial communities are in constant interaction in the rhizosphere (Bourceret et al., 2016; Akyol et al., 2019), and beside their substantial involvement in biogeochemical cycles, they have been demonstrated to behave differently in response to environmental and external factors (Bourceret et al., 2016; Foulon et al., 2016a). Notably, plant identity, mycorrhizal inoculation and the presence of pollutants in soils were demonstrated as key structuring parameters, responsible for shaping the fungal community (Bourceret et al., 2016; Akyol et al., 2019; Dagher et al., 2019). Besides, a long-term *in situ* monitoring aiming at evaluating the soil functioning as well as its productivity and promoting the ecosystem function, could provide additional information toward an optimization of the method.

## Data Availability Statement

The original contributions presented in the study are publicly available. This data can be found in NCBI, under accession PRJNA648983.

## Author Contributions

RR, JF, MH, and AL-HS contributed to conceptualization, methodology, and validation and wrote, reviewed, and edited the manuscript. RR wrote the original draft preparation. AL-HS contributed to supervision. All authors have read and agreed to the published version of the manuscript.

## Conflict of Interest

The authors declare that the research was conducted in the absence of any commercial or financial relationships that could be construed as a potential conflict of interest.

## Footnotes

1 https://benjjneb.github.io/dada2/tutorial.html
