# Supplementary material for: The Aromatic Plant Clary Sage Shaped Bacterial Communities in the Roots and in the Trace Element-Contaminated Soil More Than Mycorrhizal Inoculation – A Two-Year Monitoring Field Trial
Source: Front Microbiol. 2020 Dec 2;11:586050. doi: 10.3389/fmicb.2020.586050 (PMC7794003; doi:10.3389/fmicb.2020.586050)
Supplement: Supplementary file 1 [file Data_Sheet_1.docx]

Table S1: Descriptive results of Illumina MiSeq sequencing, followed by the step-by-step bioinformatic processing.

|  | Bacteria-soil | Bacteria-root |
| --- | --- | --- |
| No sequences (sum) | 1794423 | 1332221 |
| No sequences (mean) | 71777 | 66611 |
| No seq. filtered (mean) | 52312 | 49617 |
| No seq. merged (mean) | 27053 | 39682 |
| No seq. merg. no chimeras (mean) | 21770 | 25415 |
| No samples | 25 | 20 |
| No ASV (sum) | 4691 | 2728 |
| ASV per sample (mean) | 1261 | 516 |


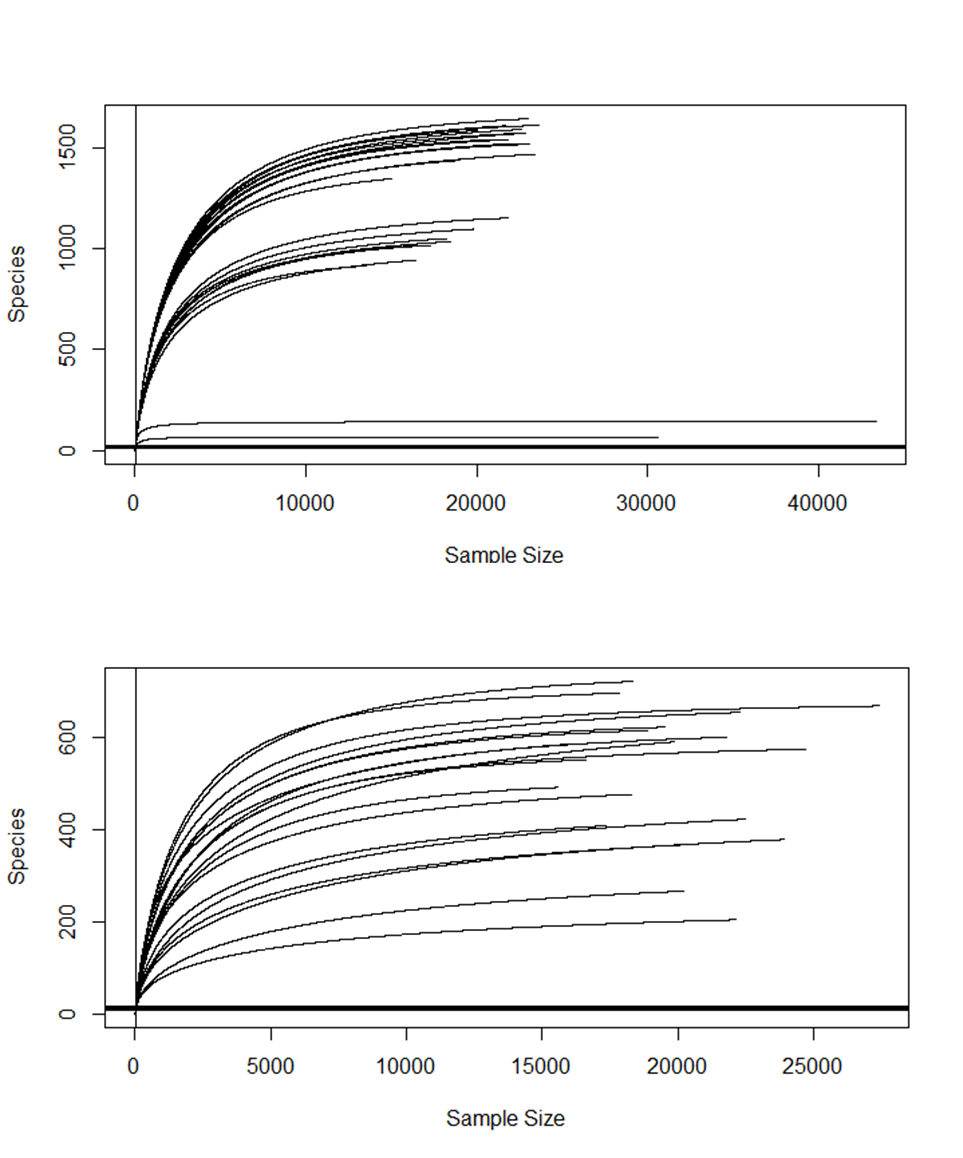


B.

A.

Figure S1: Rarefaction curves obtained for the soil (A.) and root (B.) biotopes, involving respectively 25 and 20 samples.


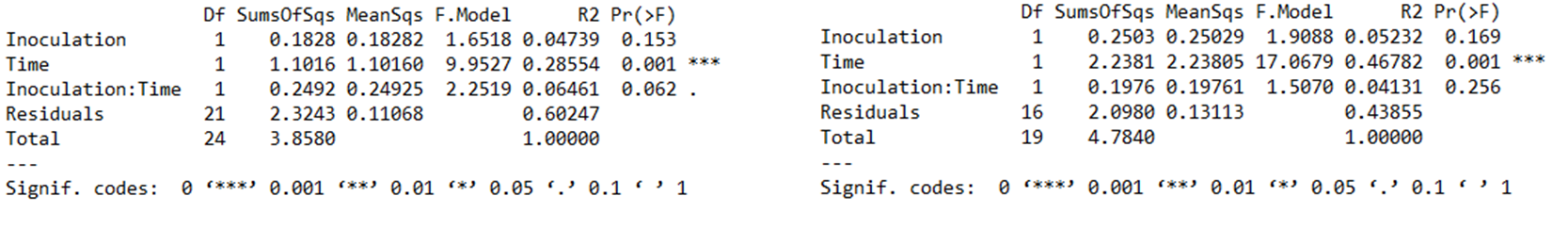


B.

A.

Figure S2: Results from the PERMANOVA analysis, generated using a model constrained for inoculation and time parameters, for soil (A.) and root (B.) bacterial communities.

Table S2: Results from Kruskall-Wallis and Tukey HSD tests for the relative abundances of the 25 most represented bacterial genera in the different experimental conditions, for both soil and root biotopes. NI: non-inoculated; I: inoculated.

|  | Soil | | | | | | Roots | | | | | | |
| --- | --- | --- | --- | --- | --- | --- | --- | --- | --- | --- | --- | --- | --- |
|  |  | **Year 1** | | **Year 2** | | | **Year 1** | | | | **Year 2** | | |
| Genus | **Initial state** | **NI** | **I** | **NI** | | **I** | **NI** | **I** | | | **NI** | **I** | |
| *Actinoplanes* | a | a | a | a | a | | ab | | ab | b | | | b |
| *Aeromicrobium* | a | a | a | a | a | | ab | | ab | b | | | b |
| *Allorhizobium-Neorhizobium-Pararhizobium-Rhizobium* | a | a | a | a | a | | ab | | ab | b | | | b |
| *Bacillus* | ab | ab | ab | b | b | | a | | a | a | | | a |
| *Blastococcus* | b | b | b | b | b | | a | | a | a | | | a |
| *Clostridium sensu stricto 1* | a | a | a | a | a | | d | | d | ab | | | ab |
| *Corynebacterium 1* | a | a | a | b | a | | a | | a | a | | | a |
| *Gaiella* | b | b | b | bc | bc | | a | | a | a | | | a |
| *Lawsonella* | a | a | ab | c | ab | | a | | a | a | | | a |
| *Lelliottia* | a | a | a | a | a | | d | | c | ab | | | ab |
| *Luedemannella* | b | b | b | b | ab | | a | | a | a | | | a |
| *Marmoricola* | ab | b | b | b | b | | a | | a | a | | | a |
| *Massilia* | a | a | a | a | a | | bc | | c | ab | | | ab |
| *Mycobacterium* | b | b | b | b | b | | a | | a | a | | | a |
| NA | e | e | e | d | d | | b | | b | c | | | c |
| *Nocardioides* | b | b | b | b | b | | ab | | ab | b | | | b |
| *Novosphingobium* | a | a | a | a | a | | ab | | bc | b | | | b |
| Others | e | e | e | e | e | | cd | | de | de | | | de |
| *Promicromonospora* | a | a | a | a | a | | ab | | ab | bc | | | b |
| *Pseudarthrobacter* | b | b | b | b | bc | | ab | | ab | c | | | c |
| *Pseudomonas* | a | a | a | a | a | | c | | c | ab | | | b |
| *Pseudonocardia* | b | b | b | b | bc | | a | | a | a | | | a |
| *Rahnella* | a | a | a | a | a | | bc | | ab | ab | | | a |
| *Skermanella* | ab | b | b | b | b | | a | | a | a | | | a |
| *Sphingomonas* | bc | b | b | bc | b | | ab | | ab | b | | | b |
| *Streptomyces* | ab | b | b | b | b | | ab | | bc | e | | | e |
